# Knowledge and Beliefs Regarding Thalassemia in an Urban Population

**DOI:** 10.7759/cureus.5268

**Published:** 2019-07-29

**Authors:** Sidra Ebrahim, Anum Z Raza, Mahnoor Hussain, Arsalan Khan, Lavita Kumari, Ramsha Rasheed, Saad Mahmood, Muneeza A Khatri, Manaal Bijoora, Ramsha Zaheer, Naveed Sattar, Wafa Sohail, Hareem Zakir, Fatima H Jafry, Areeba Memon, Shayan Anwer, Kaneez Fatima

**Affiliations:** 1 Internal Medicine, Dow Medical College and Civil Hospital, Karachi, PAK; 2 Surgery, Jinnah Medical and Dental College, Karachi, PAK; 3 Internal Medicine, Dow Medical College, Dow University of Health Sciences, Karachi, PAK

**Keywords:** thalassemia, demographics, knowledge, practices, urban, pakistan, genetics, southeast asian region

## Abstract

Background: Thalassemia is one of the most common genetic blood disorders in Asia. Consanguineous marriages and avoiding pre-marital and antenatal screening are common in Pakistan due to psychosocial, cultural, and religious factors. Few studies have investigated the beliefs regarding thalassemia, especially in a developing country. The aim of this study was to assess the knowledge, beliefs, and practices regarding thalassemia in an urban population.

Method: A cross-sectional descriptive study was conducted in the urban areas of Karachi, Pakistan over a period of six months during March 2016 through August 2016. Participants selected by representative sampling were interviewed face-to-face using a pre-designed, pre-tested questionnaire. The questionnaire was divided into four parts. The first part inquired about general demographic variables, while the second part assessed knowledge of the participant regarding thalassemia. The third and fourth parts were concerning the beliefs and practices regarding thalassemia. Data were entered and analyzed using the Statistical Package for Social Sciences (SPSS) Statistics, v. 24.0 (IBM SPSS Statistics, Armonk, NY).

Results: Only 53% (n = 720) of the respondents had heard about thalassemia. The mean knowledge score was 5.8. The total possible score ranged between 0 - 12 with the higher scores indicating better knowledge. About three-quarters (70%) of the sample did not know that an individual can be a carrier of thalassemia. Less than half (38%) of the participants viewed premarital screening for thalassemia as necessary, with only 10% agreeing that thalassemia carriers should not marry. There was no pre-marriage counseling done in 98% of the families.

Conclusion: Our study highlights the prevalence of numerous myths and a low level of knowledge regarding thalassemia in an urban population of Pakistan.

## Introduction

Thalassemia is the most commonly inherited hemolytic anemia globally [1]. Thalassemia is a heterogeneous group of inherited disorders of hemoglobin synthesis resulting in life-threatening anemia requiring regular blood transfusions [2]. It can present with a wide array of signs and symptoms, including anemia, frequent infections, poor appetite, congestive heart failure, bone deformities, failure to thrive, iron overload, and splenomegaly depending on the type and clinical severity.

According to the World Health Organization (WHO), thalassemia is found across 60 countries with a carrier population of up to 50 million. Pakistan is one of the leading South Asian countries that carry a significant burden of hemoglobinopathies, particularly beta-thalassemia. Thalassemia minor, which is the milder form of the disease, has a prevalence rate of 5% - 7% in the population [3]. Also, it is estimated that 100,000 patients suffering from thalassemia major reside in the country and every year this number is increasing by 5,000 to 9,000 new patients [4].

The treatment options include repeated blood transfusions or permanent therapy, i.e., bone marrow transplantation. Blood transfusions, besides being very inconvenient for the patient, have their own serious complications, while bone marrow transplantation is an unaffordable option for the majority in a developing country. Thus, the only way out of this socioeconomic burden is prevention. However, even in the face of imminent adverse outcomes, couples avoid genetic testing because of financial constraints, emotional distress, and fear of social stigma [5-7]. The apparent lack of awareness of the role of consanguineous marriages, along with ignorance, reluctance to undergo premarital and antenatal screening, and reluctance to terminate a pregnancy after diagnosis due to psychosocial, cultural, and religious convictions, hinders the effective prevention of the disease [8-9]. Other factors recognized are low income and a high birth rate [10].

Studies on knowledge, attitudes, and practices related to thalassemia are relatively scarce in Pakistan. Therefore, considering the above-mentioned factors, this study primarily aims at assessing the awareness, attitude, and beliefs regarding thalassemia in the urban population of Karachi to formulate a comprehensive and thorough approach to increase knowledge and cultivate an affirmative perspective towards the prevention of thalassemia in our community.

## Materials and methods

This was a community-based, cross-sectional descriptive study conducted in the urban areas of Karachi, Pakistan over a period of six months during March 2016 through August 2016. The population for the study included the general public of the city and subjects were picked out using representative sampling. Any individuals (regardless of the sex and age 18 years or above) who gave consent were included in the study. The purpose of the study was well explained to all the participants and an interview was conducted using a pre-designed, pre-tested questionnaire after taking written consent.

The interview was conducted by two interviewers who were given training on how to ask the questions in order to eliminate any chances of interviewer bias. The interviewers chosen were fluent in English, Urdu, and other regional languages to eradicate any possible miscommunication and to ensure that all eligible participants were interviewed. Furthermore, the consent form and questionnaire were translated into the national language and Urdu as well. The questionnaire was comprised of a total of 27 questions and was divided into four parts. The first part consisted of general demographic variables, which included age, sex, marital status, education, family history of thalassemia, profession, and income. The second part assessed the knowledge of the participant regarding thalassemia and contained a total of 12 questions about the nature and types of thalassemia, the role of consanguinity, carrier state, diagnosis, and treatment. The third and fourth parts contained questions concerning the beliefs and practices regarding thalassemia.

A total of 1,350 people were approached to participate in the study, out of which 40 refused or left the interview incomplete; therefore, the cooperation rate was 97%. Data were entered and analyzed using the Statistical Package for Social Sciences (SPSS) Statistics, v. 24.0 (IBM SPSS Statistics, Armonk, NY). Frequencies and percentages were computed for categorical responses. Mean and standard deviations (SD) were calculated for continuous variables.

## Results

A total of 1,350 people were approached out of which 720 (53.3%) had heard about thalassemia. These 720 individuals were included in our study. Table 1 shows the demographic characteristics of the study population. The mean age of the population was 40.3 ± 10.5 years. Most of our participants were males (64.2%) belonging to low-income families who were married (71.3%), undergraduates, unemployed (54.2%), and with no family history of thalassemia (82.4%).

**Table 1:** Baseline Demographics SD: standard deviation

	(N)	n = %
Mean age ± SD (years)	40.3 (10.5)	
Male	462	64.20%
Married	513	71.30%
Graduates	206	28.60%
Family history of thalassemia	127	17.60%
Housewives/non-employed	390	54.20%
High income status	341	47.40%

Table 2 shows the responses of participants to knowledge questions regarding thalassemia. The total possible score ranged between 0 - 12 with higher scores indicating better knowledge. The study population with scores of 7 (60%) and above were considered as having adequate knowledge, while the mean score of 5.8 of our participants indicated inadequate knowledge about thalassemia. About 40% of the participants knew that thalassemia is a genetic disease and an even greater percentage of participants knew that the disease could be diagnosed by blood tests and the need for repeated blood transfusions in cases of the major variants. About half of the participants had knowledge about the role of consanguineous marriage. The unknown part of the information consisted mainly of curability (28.89%) and the carrier state, with 70% of the sample not knowing that a person can be a carrier of thalassemia. Around 60% believed that a carrier could develop thalassemia major later on. Moreover, 92% were unaware of the fact that there are alpha and beta types of thalassemia. An overwhelming majority (84%) disagreed that if both parents are carriers, then a prenatal diagnosis should be done.

**Table 2:** Knowledge Regarding Thalassemia

Variable	No. of “yes” responses(N)	n = %
Thalassemia is a genetic disease	294	40.8%
A person can be a carrier of thalassemia	213	29.6%
An individual can have alpha or beta thalassemia	57	7.9%
Blood tests can be used for diagnosis	639	88.8%
If both partners are carriers, prenatal diagnosis should be made	115	16.0%
Beta thalassemia major require blood transfusions	303	42.1%
Thalassemia major patients are mentally ill	278	38.6%
Life expectancy of a carrier is normal	255	35.4%
Carrier can develop thalassemia major later on	412	57.2%
Consanguineous marriage has a role in thalassemia	287	39.9%
Thalassemia is curable	512	71.1%
Carrier has no symptoms	86	11.9%

Tables 3 and 4 shows the responses of participants regarding the beliefs and practices of thalassemia. About one-third of the subjects had a consanguineous marriage within the family. Less than half (38%) of the participants viewed premarital screening for thalassemia as necessary with only 10% agreeing that thalassemia carriers should not marry. There was no pre-marriage counseling done in 98% of the families, revealing the poor beliefs and practices carried out in our setup. About 10% were of the opinion that thalassemia married couples should not have children.

**Table 3:** Beliefs Regarding Thalassemia

Variable	Number of “yes” responses (N)	n = %
Thalassemia carriers should not marry	74	10.3%
Thalassemia married couples should not have children	51	7.1%
Pregnancy with thalassemia major should be terminated	16	2.2%
Will donate blood for thalassemia patients	699	97.1%
Testing for thalassemia before marriage	278	38.6%

**Table 4:** Practices Regarding Thalassemia

Variable	Number of “Yes” responses (N)	n = %
Any consanguineous marriage within family	262	36.4%
Donated blood for thalassemia patients	167	23.2%
Pre-marriage counseling done in family members	15	2.1%

## Discussion

In comparison with previous studies, the results of our study demonstrate that only 53% of respondents had heard about thalassemia. Armeli et al. showed that 85% of their respondents had heard of thalassemia, while it was 65% for a Bahrain study [11-12]. This, along with a mean knowledge score of 5.8 out of a possible 12, reflects a general lack of knowledge among the urban population. These results are alarming as Pakistan lies in the thalassemia belt with 9.8 million carriers and over 5,000 annual thalassemia births.

Our study highlights the prevalence of numerous myths about carriers and the low level of knowledge regarding the different types of thalassemia among participants. These findings are in contrast with the Malaysian study where 57% of the participants knew correctly about carriers [13]. These common misbeliefs have serious emotional effects on the carriers who tend to face many social challenges, such as difficulty in marriages and isolation. Therefore, it is very important to dispel these misconceptions and disseminate information about various types of thalassemia so that thalassemia carriers do not face stigma in society.

The most concerning finding of this study was that most of the respondents were unaware of the genetic nature (60%) and role of consanguineous marriages in disease transmission (60%). This is in line with a study from Lahore by Ishaq et al. [14]. However, in a study by Basu, 60% knew the inherited nature [15]. Owing to the complex inheritance pattern, it may be tough to explain this to the less educated masses [8]. As the studies suggest, the use of clear illustrations, audiovisual aids, and personal experience sharing can help in conveying this vital information [7, 16]. Furthermore, in the absence of perceived risk, consanguineous marriages were practiced in 36% of the participants’ families, while in comparison, the rate of consanguineous marriages was as low as 4% in the Kolkata study and as high as 82% in the Lahore study [15, 17]. Notably, only a few people believed that premarital testing for thalassemia was important, while the practice of premarital testing was merely 2%. Fear of being stigmatized in the case of a positive result and religious values are believed to have an impact on screening decisions [9]. These findings are important and can have a profound impact on our society, where consanguineous marriages are culturally preferred and premarital screening is not the norm. A study by Majeed et al. showed that on screening, 61% of thalassemia patients had carriers in their extended families [17]. In contrast, Ahmed et al. reported a lower percentage of 31% [5]. Nevertheless, the risk of the presence of asymptomatic carriers and the birth of affected children in families with thalassemia patients remains high. Since these practices are deeply embedded in our society, it will take a lot of time to end them. In this situation, promoting awareness and encouraging premarital screening can greatly help in reducing the transmission of the disease.

Another alarming finding of our study was that few people (15%) knew the importance of antenatal screening in cases in which both parents are carriers. This knowledge deficit, in the presence of millions of carriers, is alarming. Our data also revealed that only 2.3% of participants were in favor of the termination of a pregnancy if the offspring was a known thalassemia major case. This is in contrast with most other studies, including a Thailand study (88%), a Myanmar study (70%), and a Malaysian study (36%) where participants believed that termination was better than the lifelong suffering of the child [13-14, 18]. In another study, all positively screened couples opted for termination [19]. Reasons for refusal in our setup should be explored in-depth in future research, although past studies have shown an association of a complex web of moral, cultural, and religious values with a low-level of abortion acceptability [9, 20-21]. Accordingly, in communities where termination of pregnancy and antenatal screening is not socially acceptable, early carrier screening and premarital screening may be a better option than antenatal screening. Alternatively, in the presence of limited resources, studying the first child in extended families with a diagnosis can offer an economical way of population screening. This can help to identify present and future couples at risk of producing an affected child.

Regarding the diagnosis by blood tests, 89% had the correct knowledge. This is more than the Thailand study (45%) and the Lahore study (33%) which may be due to increasing awareness programs over the past 10 years [4, 22]. Surprisingly, only 9.4% of the respondents knew that blood transfusion is the treatment of thalassemia major. This is much lower than other studies, including the Kolkata study where two-thirds of the study population knew correctly [15]. A study assessing parents’ knowledge about thalassemia revealed 100% positive response to this question most certainly because they are the caretakers of a thalassemia child [23]. Despite the lack of knowledge, 97% were willing to donate blood to transfusion-dependent thalassemia patients, while 23% had already donated at some point in their life. This finding gives hope that future awareness programs might easily increase the number of blood donors by ending the myths associated with blood donation.

The results of our study further demonstrate that despite the lack of knowledge about thalassemia, the attitude of participants towards the disease was largely positive. This is consistent with findings by Pauisri et al., Srivastava et al., and Miri-Moghaddam et al. [22, 24-25]. Ninety percent of our participants believed that thalassemia patients should marry and 93% believed that they should have a child. However, in a study done in Bengal, very few had the attitude of marrying a carrier [24].

We recognized a few limitations in our study. Firstly, all data of this research were collected via face-to-face interviews; thus, responses might not be accurate due to the social desirability factor. Secondly, we did not individually assess the level of awareness in the participants according to their demographic characteristics. This warrants the need for further research which identifies the difference in knowledge on the basis of gender, education, marital status, and socioeconomic status. This will help in highlighting the demographic group which requires the highest awareness. Despite these methodological caveats, a representative sample comprising of participants from different demographic backgrounds suggest that these findings reflect the true urban trend.

## Conclusions

It was seen in our study that there is an inadequate knowledge about thalassemia. Pakistan, being a resource-constrained country, cannot afford the huge cost of curative and maintenance services for the increasing number of thalassemia patients. Subsequently, prevention remains the only viable long-term option. It has been proved by studies that community health education and outreach programs are effective in controlling the prevalence of the disease. Therefore, the development of a comprehensive prevention program that includes premarital counseling, genetic analysis, antenatal screening, and community-based awareness programs might reduce the disease burden. Our study has specifically identified the knowledge deficits that should be especially addressed in these programs.
